# Investigating social inequalities in older adults’ dentition and the role of dental service use in 14 European countries

**DOI:** 10.1007/s10198-016-0866-2

**Published:** 2017-01-07

**Authors:** Jing Shen, Stefan Listl

**Affiliations:** 1 Institute of Health and Society, Newcastle University, Newcastle upon Tyne, UK; 2 Quality and Safety of Oral Care, Radboud University, Nijmegen, The Netherlands; 3 Translational Health Economics, Heidelberg University, Heidelberg, Germany

**Keywords:** Oral health inequality, Dentition, Dental service use, Gini, Concentration index, Decomposition, Survey of Health, Ageing and Retirement in Europe (SHARE), D63, I14, I18

## Abstract

**Background:**

Oral disease, despite being largely preventable, remains the most common chronic disease worldwide and has a significant negative impact on quality of life, particularly among older adults.

**Objective:**

This study is the first to comprehensively and at a large scale (14 European countries) measure the social inequalities in the number of natural teeth (an informative oral health marker) in the over 50-year-old population and to investigate the extent to which such inequalities are attributable to dental service use.

**Methods:**

Using Wave 5 of the Survey of Health, Ageing and Retirement in Europe, which included internationally harmonized information on over 50,000 individuals across 14 European countries, we calculated Gini and Concentration indices (CI) as well as the decompositions of CIs by socioeconomic factors.

**Results:**

Sweden consistently performed the best with the lowest inequalities as measured by Gini (0.1078), CI by income (0.0392), CI by education (0.0407), and CI by wealth (0.0296). No country performed the worst in all inequality measures. However, unexpectedly, some wealthier countries (e.g., the Netherlands and Denmark) had higher degrees of inequalities than less-wealthy countries (e.g., Estonia and Slovenia). Decomposition analysis showed that income, education, and wealth contributed substantially to the inequalities, and dental service use was an important contributor even after controlling for income and wealth.

**Conclusions:**

The study highlighted the importance of comprehensively investigating oral health inequalities. The results are informative to policymakers to derive country-specific health policy recommendations to reduce oral health inequalities in the older population and also have implications for oral health improvement of the future generations.

## Introduction

Oral disease, despite being largely preventable, is still the most common chronic disease worldwide. Globally, over 3 billion people suffer from untreated dental caries [23]. Dental caries (the most common chronic condition globally [23]) and periodontal diseases (the 6th most prevalent chronic condition globally [23]) have a significant negative impact on individuals’ quality of life, particularly in middle age and older adulthood. Oral diseases are expensive to treat, and their cost to society is considerable, as it amounts to about US$ 442 billion yearly worldwide [21]. Despite the general improvement of oral health, socioeconomic inequalities in oral health persist, and remain a major concern worldwide [44], particularly among the aging population [39]. Abundant evidence documents the existence of social inequalities in oral health outcomes [1, 5, 7, 8, 30, 32, 34, 36, 40, 44]. However, few studies have examined the extent of inequalities in oral health with a consistent and objective oral health outcome measure at a large multi-country scale and even less is known about the extent to which such inequalities may be attributable to potentially modifiable risk factors such as dental care use.

This study fills the gap in the literature by measuring socioeconomic inequalities in the number of natural teeth using an internationally harmonized dataset, and examining the impact of dental service use after controlling for socioeconomic factors. The number of natural teeth (hereafter, number of teeth) is an objective and robust measure of cumulative impact of the lifetime exposure to periodontal diseases and caries. It is a relevant and comprehensive indicator of oral health, particularly in older age, because tooth loss demonstrates the accumulated impacts of adverse and beneficial risks throughout the life course [34, 36].

Regular dental attendance and dental service use have been suggested to have a positive impact on oral health [11, 27, 31, 38, 39, 42] and to be more common at the upper end of the socioeconomic scale [6, 15, 25, 26, 33, 35, 37]. It has been shown that a considerable proportion of inequalities in dental service use is established at childhood and persists throughout the entire life-course [19]. Although some evidence suggests that the association between socioeconomic status and the number of sound teeth in adults may be at least partially attributable to dental attendance patterns [9], the magnitude and distribution of inequalities in oral health and dental service use was found to be rather heterogeneous across countries [14, 19]. Moreover, it has been shown that dental non-attendance may be due to different reasons in various countries [20]. Consequently, it remains unclear whether the notion that inequalities in oral health may be attributable to inequalities in dental service use can be generalized or, more generally, whether dental service use can be considered a universal intervention point to tackle inequalities in oral health.

This study is the first to comprehensively quantify socioeconomic inequalities in the number of teeth in the over-50-years-old population at a large multi-country scale (in 14 European countries) and to investigate the extent to which such inequalities are attributable to dental service use.

## Methods

### Data

Analyses were conducted based on data from the Survey of Health, Ageing and Retirement in Europe (SHARE). SHARE is a panel survey of micro data on health, socioeconomic status, and social and family networks, comprising more than 220,000 interviews of about 110,000 individuals aged 50 or over in participating European countries [4]. SHARE samples were drawn to be representative of the older adult population (age 50+) in each country. Various types of survey sample design were used, such as simple random sampling in Sweden, and multistage sampling on the basis of regional population registers in Italy. More detailed descriptions of the SHARE methodology are available on the SHARE website http://www.share-project.org. Originated in 2004, five waves of SHARE have been collected so far (2004/05, 2006/07, 2008/09, 2010/11, and 2013). In the latest Wave 5, a new variable was added—the number of teeth, and thus we are able, for the first time, to examine the level of oral health inequalities across European countries with internationally harmonized data. Crucially, the number of teeth is a simple but informative oral health marker and represents the accumulation of disease and damage over the life course [35]. Therefore, it paints a good picture of the state of oral health. The information on number of teeth is self-reported, however, various sources of empirical evidence [10, 13, 24, 28, 29, 41] have suggested that the self-reported tooth count is a valid and reliable measure of clinical status. The fieldwork of SHARE Wave 5 started in February 2013 and was completed in November 2013 [17]. The target population of Wave 5 was individuals born in 1962 or earlier and their spouse/partner, who spoke (one of) the official language(s) of the country regardless of nationality and citizenship and who did not live abroad or in institutions. In this paper, we used release 1.0.0 (as of March 31, 2015) of SHARE Wave 5 with data from 14 European countries (Austria, Belgium, Czech Republic, Denmark, Estonia, France, Germany, Italy, Luxembourg, The Netherlands, Slovenia, Spain, Sweden, and Switzerland). While Listl and Jürges [22] provided a first descriptive overview on socioeconomic inequalities in oral health based on SHARE Wave 5 data, the present paper utilizes more sophisticated methods that provide more detailed insights into the extent and determinants of inequalities.

### Measuring inequalities

Gini index [3] and the concentration index (CI) [16] of the Lorenz curves family are used to measure the degree of oral health inequalities. The Lorenz curve for health is formed by plotting the cumulative proportion of health in the population (*y*-axis) against the cumulative population (*x*-axis) ranked by health. If the distribution of health is perfectly equal among individuals, this would plot a 45° line (perfect equality). If inequality exists, the Lorenz curve will lie between the *x*- and *y*-axes and the 45° line. The Gini coefficient is calculated by measuring the area between the Lorenz curve and the 45° line. The CI is based on a similar procedure, and the only difference with Gini is that the *x*-axis represents the cumulative proportion of the population ranked by a socioeconomic factor (in this paper, income, education, and wealth are used) rather than health. The Gini index measures *pure health inequality,* whereas the CI measures *socioeconomic factor (income, education, or wealth)-related health inequality*. Both measures range from 0 to the absolute value of 1 (+1 or −1). The value of 0 indicates complete equality where each member of the society has exactly the same level of health (in this case, the same number of teeth); the degree of inequality increases with the absolute value of the measure; and it reaches the maximum value of 1 for a society in which one member receives all the health (assuming it can be redistributed) and the rest nothing. Both CI and Gini are subject to some properties: (1) the bounds of the indices are dependent on the minimum, maximum, and mean of the health variable; (2) the value of the indices will change depending on whether health or ill-health is measured; (3) the value of the indices will not be invariant to a positive linear transformation. Those properties may potentially make cross-country and over-time comparisons troublesome. The Lorenz family indices imply that inequality remains constant if all individuals experience the same rate of improvement (a linear transformation) in health and rises only when individuals at the upper end of the health distribution improve faster than those at the bottom. However, it is also at least as plausible to say that inequality remains constant when all individuals experience the same absolute addition to their health (not necessarily proportional at the same rate, therefore, not linear transformation), which is not the case with the Lorenz family indices. A method suggested by Erreygers [12] tackles the issue by adjusting the original indices using the mean, minimum, and maximum of the health variable. We present both unadjusted and adjusted results.

### Decomposing inequalities

The observed inequalities are explained through decomposition of the concentration indices [43]. The decomposition analysis examines the contributions of different socioeconomic determinants of oral health to the overall income/education/wealth-related oral health inequalities. The decomposition captures the linear associations between the health variable and covariates. It should not be considered as a structural model or used to infer a direction of causality. A linear regression model in the form of OLS was fitted with demographic and socioeconomic factors as independent variables. The socioeconomic covariates being examined include income, education, wealth, marital status, economic activities, self-assessed health, longstanding illness, as well as age and gender. The income variable is the log transformation of annual household income. Full details of the independent variables are listed in Table 1. A range of models have been tested before selecting the model presented. The contribution of each socioeconomic factor can take both positive and negative values. When the health variable is increasing in good health as in the case of the number of teeth, a positive (negative) value indicates pro-rich (pro-poor) inequality, meaning inequality would decrease (increase) if the respective covariant was to become more equally distributed across the distribution of the socioeconomic factor in question. All the analyses are performed for each country separately and using sampling weight.

**Table 1:** Summary statistics

Country	*N* (%)	Mean number of teeth (SD)	Mean annual household net income in euros (SD)	Mean wealth in euros (SD)	Mean age in years (SD)
Continuous variables					
Austria	3507 (6.60)	17.28 (10.44)	31,876 (21,498)	228,740 (322,610)	65.79 (10.19)
Germany	4685 (8.82)	18.39 (9.69)	33,508 (21,331)	231,209 (338,949)	66.03 (10.61)
Sweden	3961 (7.46)	24.65 (6.21)	42,875 (20,647)	380,259 (434,515)	66.14 (10.13)
Netherlands	3607 (6.79)	17.59 (11.39)	34,223 (18,017)	342,646 (451,984)	64.74 (10.13)
Spain	5436 (10.24)	18.10 (10.07)	21,472 (16,989)	252,871 (422,522)	65.88 (10.86)
Italy	3972 (7.48)	18.48 (10.05)	24,865 (18,777)	257,801 (255,737)	66.54 (10.79)
France	3756 (7.07)	19.18 (9.18)	31,825 (20,117)	356,387 (457,771)	66.27 (10.40)
Denmark	3618 (6.81)	22.44 (8.74)	46,109 (23,459)	460,143 (725,167)	65.34 (9.94)
Switzerland	2274 (4.28)	22.35 (8.59)	54,439 (28,034)	651,306 (987,400)	65.63 (10.07)
Belgium	4706 (8.86)	16.94 (10.35)	32,825 (18,581)	404,359 (424,330)	65.27 (10.80)
Czech Republic	4648 (8.75)	17.59 (10.44)	14,815 (15,830)	97,238 (97,929)	64.55 (9.16)
Luxembourg	1109 (2.09)	17.87 (10.24)	59,087 (29,661)	830,037 (781,091)	64.30 (9.96)
Slovenia	2576 (4.85)	14.91 (10.27)	19,442 (17,591)	159,270 (146,548)	65.00 (10.31)
Estonia	5256 (9.90)	14.45 (9.81)	12,060 (12,533)	99,858 (121,446)	65.94 (10.11)
Total	53,111 (100)	18.67 (9.87)	30,105 (21,096)	286,899 (412,187)	65.99 (10.54)

Country	Education (pre-) primary, secondary, post-secondary	Self-assessed health (excellent, very good, good, fair, poor)	Economic activity (retired, employed, unemployed, long-term sick, homemaker/other)	Marital status (married, single, sep/div, widowed)	Respondents who had a dental visit in the past year	Male	Respondents with longstanding illness
Categorical variables							
Austria	10.11, 59.54, 30.35	8.14, 27.19, 34.97, 22.62, 7.07	62.41, 25.84, 2.23, 1.41, 8.10	63.04, 8.90, 13.27, 14.79	60.06	46.21	47.12
Germany	1.59, 65.03, 33.38	5.91, 14.46, 39.78, 30.60, 9.26	52.42, 35.19, 3.02, 2.70, 6.66	71.68, 5.48, 9.93, 12.91	79.11	46.45	60.06
Sweden	17.54, 40.69, 41.77	19.63, 26.88, 31.52, 17.31, 4.66	52.46, 43.67, 1.44, 2.01, 0.42	69.77, 8.65, 12.23, 9.35	81.63	47.93	49.51
Netherlands	10.39, 60.53, 29.08	14.16, 16.19, 42.32, 22.45, 4.88	41.62, 36.57, 2.94, 5.94, 12.93	75.88, 4.41, 8.78, 10.93	72.08	47.80	48.22
Spain	53.26, 34.71, 12.03	4.33, 16.36, 37.67, 27.52, 14.11	36.27, 26.48, 8.13, 3.97, 25.61	71.94, 7.00, 5.70, 15.36	26.99	46.03	52.52
Italy	44.87, 44.28, 10.84	7.51, 15.68, 37.64, 27.83, 11.34	48.97, 26.43, 2.69, 1.80, 20.11	73.80, 6.76, 4.00, 15.44	28.89	46.13	38.18
France	30.51, 46.61, 22.88	6.53, 16.24, 43.92, 23.54, 9.78	58.07, 29.91, 3.65, 2.85, 5.51	66.08, 8.44, 10.78, 14.70	51.16	45.79	43.65
Denmark	10.23, 47.77, 42.00	23.99, 31.81, 23.09, 16.51, 4.61	50.01, 42.16, 2.43, 3.64, 1.75	73.09, 5.65, 10.64, 10.62	83.04	48.09	49.29
Switzerland	8.75, 57.20, 34.05	11.84, 30.15, 41.81, 13.46, 2.75	44.21, 44.77, 1.40, 2.27, 7.34	63.15, 8.50, 16.59, 11.76	73.92	47.50	33.50
Belgium	16.30, 49.50, 34.20	8.42, 22.58, 43.64, 20.94, 4.41	48.46, 31.49, 3.18, 4.80, 12.08	70.37, 5.60, 11.77, 12.26	56.63	46.81	45.34
Czech Republic	5.98, 77.85, 16.17	4.91, 15.15, 40.16, 27.21, 12.56	62.39, 30.77, 2.73, 3.55, 0.56	67.91, 2.73, 15.61, 13.74	63.79	46.60	46.96
Luxembourg	37.65, 40.67, 21.69	9.63, 19.04, 38.62, 24.39, 8.32	45.77, 27.15, 1.98, 4.54, 20.55	76.54, 4.36, 8.54, 10.56	70.13	48.36	45.67
Slovenia	10.39, 62.74, 26.87	5.91, 13.40, 44.16, 25.06, 11.47	61.16, 23.19, 5.38, 1.85, 8.41	70.26, 6.46, 5.71, 17.57	44.02	46.04	41.96
Estonia	4.59, 57.51, 37.89	2.10, 4.14, 25.67, 48.06, 20.03	50.84, 37.75, 3.14, 6.37, 1.90	51.27, 9.94, 17.98, 20.82	36.77	39.13	68.78
Total	24.43, 51.77, 23.80	7.46, 16.91, 39.51, 26.33, 9.78	50.37, 31.43, 3.66, 2.69, 11.58	70.60, 6.63, 8.87, 13.91	54.81	46.35	48.81

Numbers corresponding to the percentages of each category of the variables as listed in the first row

## Results

Summary statistics of the outcome variables and socioeconomic covariates are presented in Table 1. The sample sizes for the 14 countries ranged from 1109 in Luxembourg to 5436 in Spain, which were proportional to the country sizes. The mean age for all countries is 66. On average, 46% of the sample were men for all country combined, and the percentages did not vary greatly across countries. However, there was a large disparity in other characteristics among the countries. The mean number of teeth for all countries combined was 19, whereas across countries, Estonia had the lowest average number of teeth (14) in contrast to Sweden, with the highest average number of teeth (25). This disparity also applied to the mean income and dental attendance, where large variations between countries were observed.

Luxembourg had the highest mean household annual net income (59,087 euros) as well as the highest level of total wealth (830,037 euros), whereas the lowest income was observed in Estonia (12,060 euros) and lowest total wealth observed in Czech Republic (97,238 euros); 83% of Danish reported a visit to the dentist in the last 12 months compared to 27% in Spain. The majority of the surveyed population were in good health, married, retired, and had achieved secondary education.

### Gini

Table 2 shows the Gini coefficients and the adjusted values based on Erreygers’ method. The Gini coefficients measure pure oral health inequality—the uneven distribution of oral health as measured by the number of teeth between individuals, and higher values indicate larger inequality. It is clear that the ranking order of the countries changes once Gini is adjusted using Erreygers’ method, in which original values of Gini coefficients are weighed by the mean, and minimum and maximum of the number of teeth in each country. As the observed minimum and maximum numbers of teeth are the same for all countries at 0 and 28, respectively, essentially the adjustment is solely affected by the mean number of teeth for each respective country. The positions of the top seven countries (with Sweden being the least unequal country for number of teeth) stayed the same with or without adjustment, suggesting their positions of having the least pure oral health inequality were relatively robust. A few countries moved places after the adjustment: for example, Estonia moved up five places, whereas the Netherlands moved down two places to the bottom of the rank. The reason for the difference in the direction of the movements is that while the two countries had similarly large inequality, Estonia had a smaller mean number of teeth compared to the Netherlands (observed minimum and maximum number of teeth were the same for both countries), so once Gini was adjusted, the Netherlands had a larger value than Estonia. This suggests that the Netherlands being ranked most unequal among the countries examined after adjustment is a result of the combination of relatively large Gini coefficients and generally higher attainment of teeth in the population on average, which produced a bigger spread of the teeth distribution.

**Table 2:** Gini index (measuring pure oral health inequality)

Ranking	Gini		Gini (adjusted)	
1	Sweden	0.1078	Sweden	0.3797
2	Switzerland	0.1792	Switzerland	0.5721
3	Denmark	0.1810	Denmark	0.5802
4	France	0.2592	France	0.7099
5	Germany	0.2781	Germany	0.7306
6	Italy	0.2892	Italy	0.7635
7	Spain	0.2980	Spain	0.7706
8	Luxembourg	0.3107	Estonia	0.7932
9	Austria	0.3310	Luxembourg	0.7932
10	Belgium	0.3326	Belgium	0.8050
11	Czech Republic	0.3332	Austria	0.8172
12	Netherlands	0.3456	Slovenia	0.8308
13	Estonia	0.3843	Czech Republic	0.8370
14	Slovenia	0.3899	Netherlands	0.8682

### Concentration index (CI) by income

Table 3 displays the CI by income and the adjusted values based on Erreygers’ method. CI by income measures income-related oral health inequality—how oral health as measured by number of teeth is systematically unequally distributed between individuals of different levels of income, and higher values indicate a stronger relationship between better oral health and higher income. Sweden had the lowest income-related oral health inequality both before and after adjustment, while most other countries switched positions following adjustment. Countries such as Estonia and Slovenia moved up the ranking after adjustment due to their generally lower mean number of teeth coupled with similar level of original CI, whereas other countries, for example Denmark and France, were largely dragged down by their higher mean level of tooth retention, which led to larger disparities when CI by income was weighed by the higher means. This suggests the relative position of each country’s income-related oral health inequality would be highly affected by how change occurs (linear or non-linear linear transformation) in the teeth distribution.

**Table 3:** Concentration index (CI) by income (measuring income-related oral health inequality)

Ranking	CI by income		CI by income (adjusted)	
1	Sweden	0.0392	Sweden	0.1382
2	Switzerland	0.0529	Estonia	0.1687
3	Czech Republic	0.0715	Switzerland	0.1689
4	Luxembourg	0.0717	Czech Republic	0.1797
5	Italy	0.0739	Luxembourg	0.1830
6	France	0.0765	Italy	0.1951
7	Spain	0.0765	Spain	0.1979
8	Estonia	0.0817	Slovenia	0.2071
9	Austria	0.0847	Austria	0.2090
10	Germany	0.0852	France	0.2095
11	Denmark	0.0900	Germany	0.2238
12	Slovenia	0.0972	Netherlands	0.2757
13	Netherlands	0.1097	Belgium	0.2821
14	Belgium	0.1166	Denmark	0.2883

### Concentration index (CI) by education

Table 4 displays the CI by education and the adjusted values based on Erreygers’ method. CI by education measures education-related oral health inequality—how oral health as measured by number of teeth is systematically unequally distributed between individuals of different levels of education, and higher values indicate a stronger relationship between better oral health and higher educational attainment. Sweden continued to have the lowest education-related oral health inequality both before and after adjustment, and Belgium and the Netherlands remained at the bottom of the ranks. The ranking of CI by education appears to be more robust across most countries before and after the adjustment. This shows the robustness of the relative positions of each country ranked by the degree of education-related oral health inequality that would not be affected by how change occurs (linear or non-linear transformation) in the teeth distribution.

**Table 4:** Concentration index (CI) by education (measuring education-related oral health inequality)

Ranking	CI by education		CI by education (adjusted)	
1	Sweden	0.0407	Sweden	0.1434
2	Switzerland	0.0544	Czech Republic	0.1693
3	Czech Republic	0.0674	Switzerland	0.1737
4	Denmark	0.0718	Germany	0.1944
5	Germany	0.0740	Luxembourg	0.1993
6	Luxembourg	0.0781	Italy	0.2180
7	Italy	0.0825	Denmark	0.2303
8	France	0.0891	France	0.2442
9	Austria	0.0994	Austria	0.2455
10	Spain	0.1131	Estonia	0.2479
11	Estonia	0.1201	Slovenia	0.2591
12	Slovenia	0.1216	Spain	0.2926
13	Belgium	0.1238	Belgium	0.2996
14	Netherlands	0.1295	Netherlands	0.3255

### Concentration index (CI) by wealth

Table 5 displays the CI by wealth and the adjusted values based on Erreygers’ method. This measures wealth-related oral health inequality—how oral health as measured by number of teeth is systematically unequally distributed between individuals with different levels wealth, and higher values indicate a stronger relationship between better oral health and more wealth. Sweden continued to perform best by having the lowest wealth-related oral health inequality before and after adjustment, whereas the Netherlands remained at the bottom for having the largest such inequality. The rest of the countries tend to stay within three spaces before and after the adjustment. This demonstrates the robustness of the relative positions of each country ranked by the degree of wealth-related oral health inequality that would not be affected by how change occurs (linear or non-linear transformation) in the teeth distribution.

**Table 5:** Concentration index (CI) by wealth (measuring wealth-related oral health inequality)

Ranking	CI by wealth		CI by wealth (adjusted)	
1	Sweden	0.0296	Sweden	0.1042
2	Switzerland	0.0416	Slovenia	0.1067
3	Slovenia	0.0501	Estonia	0.1202
4	Italy	0.0531	Switzerland	0.1328
5	Luxembourg	0.0564	Italy	0.1401
6	Estonia	0.0582	Luxembourg	0.1441
7	France	0.0595	France	0.1631
8	Spain	0.0657	Spain	0.1699
9	Denmark	0.0682	Czech Republic	0.1723
10	Czech Republic	0.0686	Belgium	0.1822
11	Belgium	0.0753	Austria	0.2176
12	Germany	0.0847	Denmark	0.2187
13	Austria	0.0882	Germany	0.2226
14	Netherlands	0.0918	Netherlands	0.2306

### Decompositions of concentration index (CI)

Table 6 displays the percentage contributions of each covariate in the decomposition of CI by income. Age, income, and education attainment were generally the three largest contributors to income-related oral health inequality in all countries, with varying degrees of contributions. For all countries except the Netherlands, age was the largest contributor, reflecting the cumulative nature of damage to oral health over time, despite the fact that only the over-50s population was examined, effectively reducing the age spread. Given that the CI being examined was by income, one might expect that income would contribute most to the overall income-related oral health inequality after the contribution of age, however, that was only the case for Sweden, France, Luxembourg, and Slovenia, whereas in most other countries (except the Netherlands) education was the most important factor. It is also worth noting that income contributed relatively little to the income-related oral health inequality in Spain and Czech Republic, suggesting that policy interventions relating to other factors (education, organization of oral health services) rather than income might be more effective at reducing such inequality in those countries. Dental attendance was a contributor with varying degrees of importance across countries, and it was the largest contributor for the Netherlands even ahead of age, income, and education. This suggests that increasing the rate of dental attendance would have the largest impact on reducing income-related oral health in the Netherlands. Other countries where making dental attendance more equally distributed among people with different levels of income would also make a major difference included Austria, Belgium, Czech Republic, Luxemburg, Denmark, Estonia, and Switzerland.

**Table 6:** Percentage contributions of each covariate in the decomposition of income-related oral health inequality

Variables/Country	Austria (%)	Germany (%)	Sweden (%)	Netherlands (%)	Spain (%)	Italy (%)	France (%)	Denmark (%)	Switzerland (%)	Belgium (%)	Czech (%)	Luxembourg (%)	Slovenia (%)	Estonia (%)
Age	28.44	38.39	47.55	26.74	40.31	50.58	24.04	37.99	50.13	31.37	38.49	25.35	41.86	42.47
Male	−0.54	−1.46	−2.46	−0.53	−2.12	0.72	−1.80	0.33	−1.84	−1.45	−0.67	−0.40	−0.91	−2.59
Wealth	13.45	9.18	7.53	4.05	4.09	5.33	7.14	3.48	1.63	2.46	3.61	10.14	4.95	2.39
Income	21.66	17.44	24.96	27.00	6.57	13.94	31.69	10.57	20.73	23.66	1.91	23.23	21.43	12.98
Longstanding illness	−0.45	1.71	0.86	0.94	2.75	1.55	1.98	1.21	0.57	0.50	5.96	0.51	0.63	0.09
Visited dentist in past 12 months	13.47	8.51	4.77	34.61	−1.43	−1.18	7.34	12.78	12.14	18.07	21.64	16.63	7.10	12.85
Economic status (reference: retired)														
Employed	3.22	−0.77	−2.96	5.34	5.63	7.84	−0.81	3.16	−6.63	3.66	8.43	5.92	4.76	17.42
Unemployed	0.15	0.71	0.12	−0.79	−0.42	−0.21	0.62	0.01	0.54	−0.44	0.18	0.17	−0.83	−0.08
Sick	−0.73	0.32	−0.17	−0.17	−0.03	−1.35	−0.32	0.11	1.22	0.83	−0.05	1.81	0.19	0.78
Home maker/other	1.26	0.34	0.01	0.07	0.76	−5.04	1.14	0.39	0.07	0.50	0.02	0.01	3.02	0.13
Self-assessed health (reference: excellent)														
Very good	−1.28	0.20	−0.19	−0.62	−0.06	3.32	0.12	−0.28	−0.34	−3.93	3.17	0.02	2.51	−0.01
Good	1.52	−0.78	0.25	0.29	−1.35	3.47	−0.45	0.39	1.90	1.11	1.48	−0.04	−0.30	−4.60
Fair	6.52	5.81	7.96	1.60	4.50	−0.28	5.17	2.25	4.63	5.06	−0.73	1.73	0.23	4.11
Poor	3.97	3.80	3.11	0.98	7.67	3.73	3.02	2.65	1.52	1.80	−0.61	3.76	1.72	9.62
Education attainment^a^ (reference: (pre-) primary)														
Secondary	−4.69	−7.85	−1.87	−4.98	6.42	4.52	1.15	−8.25	−9.90	−3.94	−2.75	3.89	−2.09	−5.29
Post-secondary/tertiary	17.05	19.51	21.44	22.51	11.61	10.97	18.25	27.49	23.55	23.80	10.46	11.24	15.63	16.54
Marital status (reference: married)														
Single	−5.34	−1.81	−12.83	−23.08	2.18	−7.08	−12.26	3.01	−4.40	−3.86	−2.30	−5.16	−6.53	−5.21
Sep/div	−0.27	0.23	3.01	3.31	0.45	0.34	1.72	0.05	−0.58	0.79	−0.07	−1.70	0.35	1.37
Widowed	1.96	5.11	6.14	7.19	8.99	11.38	6.59	2.82	3.96	0.39	7.73	0.55	6.48	7.11

^a^Educational attainment—three categories according to the International Standard Classification of Education (ISCED): (pre-) primary (ISCED scores 0 and 1), secondary (ISCED scores 2 and 3), and post-secondary and tertiary (ISCED scores 4–6). See [17, 18] for a detailed description of ISCED

Table 7 displays the percentage contributions of each covariate in the decomposition of CI by education. Age and education attainment were generally the largest contributors to education-related oral health inequality in all countries, with varying degrees of contributions. Education was overwhelmingly the largest contribution to the overall education-related oral health inequality in all countries except Spain. Income was no longer an important contributor to education-related oral health inequality, except in France. The Netherlands continued to have the largest contribution from dental attendance, suggesting that most dental attendance were taken up by people with higher educational attainment. Other countries where increasing dental attendance would help reduce education-related oral health inequality included Austria, Denmark, Switzerland, Belgium, Czech Republic, Luxembourg, and Estonia.

**Table 7:** Percentage contributions of each covariate in the decomposition of education-related oral health inequality

Variables/Country	Austria (%)	Germany (%)	Sweden (%)	Netherlands (%)	Spain (%)	Italy (%)	France (%)	Denmark (%)	Switzerland (%)	Belgium (%)	Czech Republic (%)	Luxembourg (%)	Slovenia (%)	Estonia (%)
Age	18.54	24.88	29.47	15.75	33.55	37.36	27.39	20.77	36.87	17.11	11.06	25.59	32.00	24.50
Male	−0.65	−3.28	0.62	−0.49	−1.04	0.73	−1.51	0.13	−2.35	−0.63	−0.67	−0.72	−0.89	0.59
Wealth	7.31	6.06	4.67	2.08	2.70	3.53	4.87	2.08	1.23	1.88	2.50	8.15	3.14	1.25
Income	4.49	6.25	8.89	9.46	1.55	3.26	10.16	4.93	5.51	8.24	0.35	7.48	5.50	2.08
Longstanding illness	−0.40	1.44	0.43	0.75	2.00	2.20	1.55	0.74	0.36	0.42	3.76	0.45	0.51	0.06
Visited dentist in past 12 months	14.06	8.40	4.18	29.83	-1.22	−1.35	7.37	15.50	12.15	16.43	19.12	16.36	6.20	13.21
Economic status (reference: retired)														
Employed	2.12	−0.48	−1.65	2.64	4.21	5.24	−0.60	1.58	−5.23	1.89	3.65	5.50	3.59	7.84
Unemployed	0.02	0.16	0.02	−0.27	0.47	−0.01	−0.33	−0.09	0.45	−0.06	0.14	0.08	−0.27	0.01
Sick	−0.29	0.65	0.01	−0.05	−0.03	−1.00	−0.13	0.16	1.08	0.57	−0.15	1.37	0.10	0.40
Home maker/other	1.56	0.72	−0.01	0.05	−0.40	−3.54	0.98	0.32	0.27	0.41	−0.01	0.02	3.35	0.12
Self-assessed health (reference: excellent)														
Very good	−1.37	0.18	−0.18	−0.50	−0.05	3.29	0.10	−0.29	−0.29	−3.63	3.63	0.03	4.30	−0.01
Good	0.64	−1.08	0.26	0.18	−1.23	2.66	−0.88	0.45	3.09	0.76	−0.36	−0.06	−0.28	−3.16
Fair	7.18	5.37	5.76	1.46	4.13	−0.13	5.41	1.72	3.19	4.64	−0.63	2.24	0.29	2.73
Poor	3.23	3.94	1.39	0.51	6.83	3.86	2.41	1.88	0.48	1.53	−0.26	3.35	1.78	7.29
Education attainment^a^ (reference: (pre-) primary)														
Secondary	−13.45	−24.71	−21.17	−9.09	12.41	16.07	3.16	−39.00	−35.37	−11.17	−9.74	7.87	−5.43	−15.29
Post-secondary/tertiary	48.95	59.81	60.13	39.65	17.37	22.28	34.41	86.91	76.87	55.01	36.48	27.69	38.14	44.41
Marital status (reference: married)														
Single	−0.90	−0.63	−2.28	−5.50	0.45	−2.51	−3.27	0.66	−1.14	−0.50	−0.54	−1.73	−2.67	−0.80
Sep/div	0.08	−0.01	−0.24	0.20	−1.45	−0.28	−0.79	−0.01	0.16	−0.19	0.01	0.06	−0.15	−0.39
Widowed	1.36	4.06	2.69	3.30	5.07	7.22	4.85	1.47	2.85	0.18	3.22	0.48	4.49	3.04

^a^Educational attainment—three categories according to the International Standard Classification of Education (ISCED): (pre-) primary (ISCED scores 0 and 1), secondary (ISCED scores 2 and 3), and post-secondary and tertiary (ISCED scores 4–6). See [17, 18] for a detailed description of ISCED

Table 8 displays the percentage contributions of each covariate in the decomposition of CI by wealth. Wealth only appeared to be the most important contributing factor to wealth-related oral health inequality in Austria, Germany, Italy, France, Luxembourg, and Slovenia. In many countries (Sweden, the Netherlands, Spain, Denmark, Belgium, Czech Republic, Estonia, and Switzerland), education continued to play an important role even after controlling for wealth and income. The Netherlands continued to have dental attendance as the largest contributor. Dental attendance was also important in countries such as Austria, Switzerland, Belgium, Luxembourg, Estonia, Czech Republic, and Denmark.

**Table 8:** Percentage contributions of each covariate in the decomposition of wealth-related oral health inequality

Variables/Country	Austria (%)	Germany (%)	Sweden (%)	The Netherlands (%)	Spain (%)	Italy (%)	France (%)	Denmark (%)	Switzerland (%)	Belgium (%)	Czech (%)	Luxembourg (%)	Slovenia (%)	Estonia (%)
Age	24.94	18.38	13.29	22.85	13.27	26.63	5.87	18.42	12.09	14.17	22.38	4.50	21.57	23.67
Male	−0.41	−0.78	−1.62	−0.45	−0.44	0.76	−1.27	0.30	−1.21	−1.56	−0.30	−0.16	−0.76	−1.83
Wealth	31.41	21.12	23.11	10.34	14.10	21.30	23.61	10.49	6.82	10.70	10.52	31.78	29.56	10.63
Income	7.76	7.88	14.77	15.55	2.47	5.96	17.40	6.63	7.12	13.55	0.55	11.18	12.68	5.49
Longstanding illness	−0.50	1.89	0.54	1.33	2.14	2.99	2.20	1.57	0.48	0.86	4.61	0.27	0.53	0.08
Visited dentist in past 12 months	16.19	9.88	10.24	40.05	−1.02	−1.98	7.92	19.98	21.20	22.37	25.11	25.39	11.90	19.18
Economic status (reference: retired)														
Employed	2.01	−0.38	−0.69	3.91	4.09	3.03	−0.17	1.71	−2.47	1.78	4.63	2.85	1.82	11.23
Unemployed	0.15	1.14	0.19	−1.08	−1.57	−0.32	0.75	−0.17	1.37	−0.79	0.17	0.26	−1.82	−0.15
Sick	−0.85	1.19	−0.12	−0.20	−0.07	−2.03	−0.45	0.23	2.46	2.33	−0.14	2.43	0.50	0.70
Home maker/other	0.29	−0.31	−0.04	0.00	0.30	−3.76	−0.11	0.12	−0.27	0.13	−0.02	−0.01	2.88	0.15
Self-assessed health (reference: excellent)														
Very good	−0.96	0.17	−0.15	−0.56	−0.07	2.04	0.15	−0.31	−0.53	−5.40	2.37	0.01	7.61	−0.01
Good	−0.25	−0.87	0.22	0.50	−1.53	3.06	−0.20	0.34	3.22	−0.38	1.21	−0.08	0.19	−3.91
Fair	5.21	4.57	7.14	1.83	6.29	−0.16	5.76	2.54	7.26	8.14	−0.56	1.85	0.22	3.19
Poor	6.34	4.64	2.01	1.13	8.15	3.49	3.29	2.51	1.78	2.82	−0.44	2.52	2.49	9.45
Education attainment^a^ (reference: (pre-) primary)														
Secondary	−2.97	−5.26	−8.58	−2.56	5.86	3.27	−0.26	−9.21	−11.31	−3.19	−3.11	9.29	−3.33	−8.08
Post-secondary/tertiary	13.83	13.85	24.12	17.88	11.60	12.48	18.80	26.42	27.78	27.86	11.25	10.58	23.77	22.82
Marital status (reference: married)														
Single	−4.01	−1.30	−9.92	−24.14	1.51	−6.14	−10.05	2.59	−3.77	−3.75	−1.61	−5.50	−10.86	−3.65
Sep/div	−0.19	0.29	3.06	4.47	0.26	0.56	1.82	0.07	−1.30	1.43	−0.09	−2.16	0.93	1.51
Widowed	1.67	3.07	4.15	5.48	5.87	8.58	4.59	2.06	1.87	0.28	5.34	0.46	8.71	5.27

^a^Educational attainment—three categories according to the International Standard Classification of Education (ISCED): (pre-) primary (ISCED scores 0 and 1), secondary (ISCED scores 2 and 3), and post-secondary and tertiary (ISCED scores 4–6). See [17, 18] for a detailed description of ISCED

## Discussion

This study is the first to comprehensively quantify socioeconomic inequalities in oral health (measured by the number of teeth) in the over-50s population from 14 European countries and to examine the extent to which such inequalities are attributable to dental service use. We measured pure, and income-, education-, and wealth-related inequalities in oral health. We also examined the contributions of socioeconomic factors to income-, education-, and wealth-related oral health inequalities through decompositions. When calculating the degrees of inequality, adjustments were made to take into consideration the mean, minimum, and maximum of the oral health variable in each country. That is because the indices derived from the Lorenz family are not scale-invariant. Among all countries, Sweden consistently remained the best-performing country with lowest Gini (0.110), CI by income (0.039), CI by education (0.041), and CI by wealth (0.030). No single country performed the worst for all three inequality measures. However, surprisingly, some wealthier European countries (e.g., the Netherlands and Denmark) had higher degrees of inequalities compared to less-wealthy European countries (e.g., Estonia and Slovenia). Further decomposition analysis showed that apart from age (which is recognized as an important factor in dentition), considerable proportions of inequality were attributable to dental attendance, even after controlling for income, wealth, and education, in the Netherlands, Austria, Belgium, Czech Republic, Luxembourg, Denmark, and Switzerland.

We found that inequalities in oral health existed in all countries, and the degree of which varied significantly across countries. In terms of pure oral health inequality, the Gini coefficient of the country at the bottom of the rank was almost four times larger than that of the country at the top of the rank. For income-, education-, or wealth-related oral health inequalities, the country at the bottom of the rank had a CI value three times larger than the country at the top. Once adjustments were applied, the relative gap between countries became smaller, and the ranking orders changed. This reflects the nature of the inequality tool, as Gini and CI from the Lorenz family are sensitive to the transformations of the health variable. After the adjustment was applied, because the recorded minimum and maximum number of teeth are 0 and 28, respectively, for all countries, essentially the cause of the changes in rankings between original Gini and CI values and the adjusted ones is the difference in the mean number of teeth between countries. This happens especially among the countries with similar original Gini and CI values, but presenting large differences in the mean of the health variable. In terms of pure health inequality, the obvious examples are the Netherlands and Estonia. They both had very similar levels of inequality according to the original Gini coefficients, but the Netherlands had a relatively larger mean number of teeth, therefore, after the Gini coefficients were adjusted, the Netherlands had a higher adjusted inequality score than Estonia. Similar switches of places were also observed among some other countries and in the case of CIs.

The changes in rankings among countries between the adjusted and unadjusted inequality measures show the robustness of the relative position of each country ranked by the degree of oral health inequalities. Sweden was proven to be robust in its low degree of oral health inequalities, as its position stayed at the top of the rankings regardless of the adjustment; therefore, it is safe to say that Sweden was the best-performing country with the lowest degrees of inequalities that were not sensitive to any changes in the mean of the number of teeth variable and how change occurs in the teeth distribution. In contrast, the other Scandinavian country included in the survey—Denmark, had a very different outlook. Denmark performed relatively well in terms of pure health inequality as measured by Gini, however, it appeared to have one of the worst income-related oral health inequalities—the distribution of oral health was highly correlated with the distribution of income, and became the worst once the adjustment was applied. When comparing the relatively wealthier Western European countries and the less-affluent Eastern European countries, some of the former performed worse, in particular, the case of Belgium and the Netherlands. They took the bottom two places in the ranking for both CI by income and CI by education (except in the adjusted CI by income ranking, they both went up one place above Denmark). The Netherlands also stayed at the bottom of the ranking for CI by wealth. This suggests that although they had a relatively higher overall mean number of teeth, the distribution of which was highly correlated with the income, education, and wealth distribution in the two countries. Additionally, the Netherlands had a high pure oral health inequality reflected by adjusted Gini.

The stark differences in oral health inequalities across European countries, especially among those with similar social security and health care systems, may suggest various pathways that lead to inequalities. After further investigation through the decomposition analyses, a number of factors were shown to be major contributions of socioeconomic-related inequalities. Education remained an important contributor in most countries, regardless of whether the CIs were calculated by income, education, or wealth. This may suggest oral health outcomes may be largely determined by how efficiently individuals produce good oral health (e.g., through perception of dental service, self-maintenance of oral health, knowledge of oral health prevention). Income appeared to have contributed more to oral health inequalities in wealthier countries than in less-affluent countries, which may subscribe to the relative income hypothesis [45]. Having controlled for socioeconomic factors, dental attendance still showed a varying but important contribution to the overall levels of inequality.

Conceptually, health care use is thought of being related to need (including perceived need), predisposing (e.g., age, sex), enabling (e.g., income), and system-level (e.g., healthcare delivery and organization) factors [2]. A recent study based on SHARE data found that the highest proportion of respondents without any regular dental attendance throughout their lifetime was from the Southern welfare-state regime, followed by the Eastern, the Bismarckian, and the Scandinavian welfare-state regimes [20]; As for reasons of non-attendance, factors such as patients’ perception (perceived need) that regular dental treatment is ‘not necessary’ or ‘not usual’ were identified to be the predominant reason for non-attendance in all these welfare-state regimes, “not affordable” (enabling factor) and “No provider nearby” (healthcare delivery and organization factor) were also cited with different prominence across countries [20]. In this study, dental attendance was consistently one of the largest contributors to oral health inequalities in the Netherlands, followed by other Bismarckian and Eastern welfare-state countries, suggesting that increasing the rate of dental attendance would have great impact on reducing socioeconomic inequalities in oral health. Combining these findings with previous evidence on the reasons for dental non-attendance [20], it seems sensible to argue in favor of differential prioritization of intervention points for health policy in the various countries. For example, policies aiming at oral health promotion (such as raising awareness for the benefits of regular dental attendance) may have a larger impact in reducing inequalities among those disadvantaged in Bismarckian and Eastern welfare-state countries than in Scandinavia. This is also supported by the finding that levels of education consistently remained an important contributor to the inequalities, so that raising awareness and knowledge of good oral health practice would be effective at reducing socioeconomic inequalities in oral health.

Given that the study population is individuals over 50 years old, which is a more homogeneous population than the general population, and as oral health is measured by number of teeth, one might expect a more equal distribution as tooth retention is significantly associated with age; however, our results show a different picture. The situation with the older generation may be a key indicator of how different health systems have performed and what lessons we can learn to improve oral health and reduce gaps between individuals for the future generations.

The strength of this paper lies in the use of a unique and large-scale dataset that is harmonized across countries, and being able to investigate the role of dental service use after controlling for socioeconomic confounders (income, education, and wealth). For the first time, a clinically important marker of oral health is collected in a large-scale multi-country survey, so that oral health inequalities can be consistently examined across European countries. This allows researchers and policymakers the opportunity to conduct cross-country comparisons and benchmark the performances of countries against each other as well as longitudinally for future comparisons on any health improvement. There are, however, limitations to this study. The variable on whether respondents visited a dentist in the last 12 months represents the respondents’ current dental attendance status, whereas tooth loss is an accumulation of risks over the entire previous lifetime. Additionally, the dental visit variable did not specify whether it was prevention or treatment visits. Nevertheless, it is reasonable to assume continuity in dental attendance behavior and it has been shown that a considerable proportion of inequalities in dental care use was established at childhood and persisted throughout an individual’s entire life course [18].

## Conclusions

In light of the stark differences in oral health inequalities among European countries, and how dental service use (measured by dental visits) contributes differently to the measured inequalities, the study highlights the importance of investigating distributional issues in older populations’ oral health. These results may be useful to derive country-specific health policy recommendations about the relevance of improving dental care use in order to reduce inequalities against the background of population ageing. The study also provides benchmarks for future comparisons of inequalities in oral health and lessons can be learnt to improve oral health and reduce gaps between individuals for future generations.

## References

[1] Aida J, Kondo K, Kondo N, Watt RG, Sheiham A, Tsakos G (2011). Income inequality, social capital and self-rated health and dental status in older Japanese. Soc Sci Med.

[2] Andersen, R.M.: Behavioral model of families’ use of health services. Chicago: Center for Health Administration Studies, University of Chicago (1968)

[3] Atkinson AB (1970). On the measurement of inequality. J Econ Theory.

[4] Börsch-Supan A, Brandt M, Hunkler C, Kneip T, Korbmacher J, Malter F (2013). Data resource profile: the survey of health, ageing and retirement in Europe (SHARE). Int J Epidemiol.

[5] Costa SM, Martins CC, Bonfim Mde L, Zina LG, Paiva SM, Pordeus IA, Abreu MH (2012). A systematic review of socio-economic indicators and dental caries in adults. Int J Environ Res Public Health.

[6] Crocombe LA, Broadbent JM, Thomson WM, Brennan DS, Slade GD, Poulton R (2011). Dental visiting trajectory patterns and their antecedents. J Public Health Dent.

[7] Do LG (2012). Distribution of caries in children: variations between and within populations. J Dent Res.

[8] Do LG, Spencer AJ, Slade GD, Ha DH, Roberts-Thomson KF, Liu P (2010). Trend of income-related inequality of child oral health in Australia. J Dent Res.

[9] Donaldson AN, Everitt B, Newton T, Steele J, Sherriff M, Bower E (2008). The effects of social class and dental attendance on oral health. J Dent Res.

[10] Douglass CW, Berlin J, Tennstedt S (1991). The validity of self-reported oral health status in the elderly. J Public Health Dent.

[11] Dye BA, Selwitz RH (2005). The relationship between selected measure of periodontal status and demographic and behavioural risk factors. J Clin Periodontol.

[12] Erreygers G (2009). Correcting the concentration index. J Health Econ.

[13] Gilbert GH, Duncan RP, Kulley AM (1997). Validity of self-reported tooth counts during a telephone screening interview. J Public Health Dent.

[14] Guarnizo-Herreño CC, Watt RG, Fuller E, Steele JG, Shen J, Morris S, Wildman J, Tsakos G (2014). Socio-economic position and subjective oral health: findings for the adult population in England, Wales and Northern Ireland. BMC Public Health.

[15] Holst D, Grytten J, Skau I (2005). Demand for dental service and expenditures for dental treatment in the Norwegian adult population. Nor Dent J.

[16] Kakwani NC, Wagstaff A, Van Doorslaer E (1997). Socioeconomic inequalities in health: measurement, computation and statistical inference. J Econom.

[17] Kneip, T., Malter, F., Sand, G.: Fieldwork monitoring and survey participation in fifth Wave of SHARE. In: Malter, F., Börsch-Supan, A. (eds.) SHARE Wave 5: innovations & methodology. Munich, Germany: Munich Center for the Economics of Aging (MEA) at the Max Planck Institute for Social Law and Social Policy (MPISOC) (2015)

[18] Listl S (2011). Income-related inequalities in dental service utilization by Europeans aged 50+. J Dent Res..

[19] Listl S (2012). Inequalities in dental attendance throughout the life-course. J Dent Res.

[20] Listl S, Moeller J, Manski R (2014). A multi-country comparison of reasons for dental non-attendance. Eur J Oral Sci.

[21] Listl S, Galloway J, Mossey P, Marcenes W (2015). Global economic impact of dental diseases. J Dent Res.

[22] Listl, S., Jürges, H.: Social inequalities in oral health—towards targeted health policy interventions. In: Börsch-Supan, A., et al. (eds.) SHARE: a European policy device for inclusive ageing societies, DeGruyter (2015)

[23] Marcenes W, Kassebaum NJ, Bernabé E, Flaxman A, Naghavi M, Lopez A, Murray CJ (2013). Global burden of oral conditions in 1990–2010: a systematic analysis. J Dent Res.

[24] Matsui D (2017). Validity of self-reported number of teeth and oral health variables. BMC Oral Health.

[25] Murakami K, Aida J, Ohkubo T, Hashimoto H (2014). Income-related inequalities in preventive and curative dental care use among working-age Japanese adults in urban areas: a cross-sectional study. BMC Oral Health.

[26] Palencia L, Espelt A, Cornejo-Ovalle M, Borrell C (2014). Socioeconomic inequalities in the use of dental care services in Europe: what is the role of public coverage?. Community Dent Oral Epidemiol.

[27] Petersen PE, Kjoller M, Christensen LB, Krustrup U (2004). Changing dentate status of adults, use of dental health services, and achievement of national dental health goals in Denmark by the year 2000. J Public Health Dent.

[28] Pitiphat W, Garcia RI, Douglass CW, Joshipura KJ (2002). Validation of self-reported oral health measures. J Public Health Dent.

[29] Ramos RQ, Bastos JL, Peres MA (2013). Diagnostic validity of self-reported oral health outcomes in population surveys: literature review. Rev Bras Epidemiol.

[30] Sanders AE, Slade GD, Turrell G, Spencer AJ, Marcenes W (2006). The shape of the socio-economic-oral health gradient: implications for theoretical explanations. Community Dent Oral Epidemiol.

[31] Sanders AE, Spencer AJ, Slade GD (2006). Evaluating the role of dental behaviour in oral health inequalities. Community Dent Oral Epidemiol.

[32] Sanders AE, Slade GD, John MT, Steele JG, Suominen-Taipale AL, Lahti S, Nuttall NM, Allen PF (2009). A cross-national comparison of income gradients in oral health quality of life in four welfare states: application of the Korpi and Palme typology. J. Epidemiol Community Health.

[33] Seirawan H (2008). Parsimonous prediction model for the prevalence of dental visits. Community Dent Oral Epidemiol.

[34] Shen J, Wildman J, Steele J (2013). Measuring and decomposing oral health inequalities in an UK population. Community Dent Oral Epidemiol.

[35] Somkotra T, Detsomboonrat P (2009). Is there equity in oral healthcare utilization: experience after achieving Universal Coverage. Community Dent Oral Epidemiol.

[36] Steele J, Shen J, Tsakos G, Fuller E, Morris S, Watt R, Guarnizo-Herreño C, Wildman J (2015). The Interplay between socio-economic inequalities and clinical oral health. J Dent Res.

[37] Tchicaya A, Lorentz N (2014). Socioeconomic inequalities in the non-use of dental care in Europe. Int J Equity Health.

[38] Thomson WM, Williams SM, Broadbent JM, Poulton R, Locker D (2010). Long-term dental visiting patterns and adult oral health. J Dent Res.

[39] Tsakos G (2011). Inequalities in oral health of the elderly: rising to the public health challenge?. J Dent Res.

[40] Tsakos G, Demakakos P, Breeze E, Watt RG (2011). Social gradients in oral health in older adults: findings from the English longitudinal survey of aging. Am J Public Health.

[41] Ueno M, Zaitsu T, Shinada K, Ohara S, Kawaguchi Y (2010). Validity of the self-reported number of natural teeth in Japanese adults. J Investig Clin Dent.

[42] Unell L, Soederfeldt B, Halling A, Birkhed D (1999). Explanatory models for clinically determined and symptom-reported caries indicators in an adult population. Acta Odontol Scand.

[43] Wagstaff A, van Doorslaer E, Watanabe N (2003). On decomposing the causes of health sector inequalities with an application to malnutrition inequalities in Vietnam. J Econom.

[44] Watt RG, Listl S, Peres M, Heilmann A (2015). Social inequalities in oral health: from evidence to action.

[45] Wilkinson RG (1997). Socioeconomic determinants of health: health inequalities: relative or absolute material standards?. BMJ.

